# Lectures on the Malarial Fevers

**Published:** 1899-03

**Authors:** 


**Lectures on the Malarial Fevers.** By WILLIAM SYDNEY THAYER, M.D. Pp. viii., 326. New York: D. Appleton and Company. 1897.

The indiscriminate way in which the term malaria is generally applied to all sorts of febrile and afebrile conditions is an evidence of how backward medical opinion has been in appreciating recent advances in our knowledge of this subject. Professor Thayer's book will be found to contain a full, lucid, and succinct account of Paludism, the various chapters being devoted to history of the discovery of the parasite, methods of examination of the blood, description of the hæmatozoa, clinical description of the malarial fevers, sequelæ and complications, morbid anatomy, general pathology, diagnosis and treatment. A large number of illustrative charts are included, and also plates descriptive of the various phases of the parasites.

Clinically, the author recognises three varieties of malarial fever—tertian (including quotidian), quartan (including double and triple quartan), and æstivo-autumnal, these being respectively the result of infection by hæmamœba vivax, hæmamœba malariæ, and hæmatozoon falciparum. It has been customary to regard æstivo-autumnal fevers as due to a variety of organisms, but these the author is inclined to regard as a single variety of parasite. The crescentic and ovoid bodies, which have given rise to so much discussion, Professor Thayer considers are developed from the smaller forms in the ordinary cycle of development and are incapable of further development in the body. Manson's view, that the flagellated bodies represent the forms in which the malarial parasite exists outside the human body, does not meet with Professor Thayer's support, he himself tending to regard them as degenerate forms. That the mosquito acts an intermediate part he thinks is not proven. "On the whole, it must be said that we are absolutely ignorant of the form in which the malarial parasite exists outside of the human body, and equally ignorant of the manner in which it enters." In the clinical description the fact is emphasised that there may be a special localisation of the parasites in various organs, and thus the attack may take on a special type: thus, with the brain bearing the brunt, there may be coma, or bulbar paralysis, with localisation in the gastro-intestinal mucosa, choleriform symptoms may be the most prominent. The direct exciting cause of the malarial paroxysm Professor Thayer concludes is the liberation of some toxic substance by the specific parasites at the time of their sporulation. The discussion of this point, and indeed the whole chapter devoted to the general pathology, is full of the most interesting and original writing.

Under the head of "Treatment" is discussed the action of quinine on the malarial parasite, and the systematic and scientific administration of this drug is insisted upon, the writer giving it as his opinion that there is scarcely another drug in the Pharmacopœia, unless it be digitalis, which is more abused.

Professor Thayer's book promises and deserves to become a standard work on the subject of malarial fevers.

